# Deep brain stimulation in Parkinson’s disease: state of the art and future perspectives

**DOI:** 10.1590/0004-282X-ANP-2022-S133

**Published:** 2022-08-12

**Authors:** Carina França, Rafael Bernhart Carra, Juliete Melo Diniz, Renato Puppi Munhoz, Rubens Gisbert Cury

**Affiliations:** 1 Universidade de São Paulo, Faculdade de Medicina, Departamento de Neurologia, Centro de Distúrbios do Movimento, São Paulo, SP, Brazil.; 2 Universidade de São Paulo, Faculdade de Medicina, Departamento de Neurologia, Divisão de Neurocirurgia Funcional, São Paulo, SP, Brazil.; 3 University of Toronto, Toronto Western Hospital, Movement Disorders Centre, Toronto, ON, Canada.

**Keywords:** Parkinson Disease, Deep Brain Stimulation, Transcutaneous Electric Nerve Stimulation, Doença de Parkinson, Estimulação Encefálica Profunda, Estimulação Elétrica Nervosa Transcutânea

## Abstract

For more than 30 years, Deep Brain Stimulation (DBS) has been a therapeutic option for Parkinson’s disease (PD) treatment. However, this therapy is still underutilized mainly due to misinformation regarding risks and clinical outcomes. DBS can ameliorate several motor and non-motor symptoms, improving patients’ quality of life. Furthermore, most of the improvement after DBS is long-lasting and present even in advanced PD. Adequate patient selection, precise electric leads placement, and correct DBS programming are paramount for good surgical outcomes. Nonetheless, DBS still has many limitations: axial symptoms and signs, such as speech, balance and gait, do not improve to the same extent as appendicular symptoms and can even be worsened as a direct or indirect consequence of surgery and stimulation. In addition, there are still unanswered questions regarding patient’s selection, surgical planning and programming techniques, such as the role of surgicogenomics, more precise imaging-based lead placement, new brain targets, advanced programming strategies and hardware features. The net effect of these innovations should not only be to refine the beneficial effect we currently observe on selected symptoms and signs but also to improve treatment resistant facets of PD, such as axial and non-motor features. In this review, we discuss the current state of the art regarding DBS selection, implant, and programming, and explore new advances in the DBS field.

## RESUMO

Há mais de 30 anos, a Estimulação Cerebral Profunda (ECP) é uma opção de tratamento para pessoas com doença de Parkinson (DP). Apesar disso, a ECP ainda é subutilizada, em grande parte por desinformação acerca dos riscos e dos benefícios desse tratamento. A ECP melhora os sintomas motores e não motores da DP, melhorando, assim, a qualidade de vida dos pacientes. Grande parte dos benefícios gerados pela ECP têm longa duração, estando presentes até mesmo em fases avançadas da doença. A seleção adequada dos pacientes, o preciso posicionamento dos eletrodos cerebrais, e a programação correta da ECP são fundamentais para que haja benefício após a cirurgia. Todavia, existem ainda muitas limitações em relação ao tratamento com ECP. Sintomas axiais, como fala e marcha, não melhoram tanto quanto os sintomas apendiculares, e podem até mesmo piorar após a cirurgia. Existem muitas dúvidas relacionadas à seleção de pacientes, especialmente nos aspectos de imagem e genética. Em relação à questão cirúrgica, novas técnicas de imagem podem auxiliar o posicionamento correto dos eletrodos cerebrais. Novas estratégias de programação e avanços de *hardware* podem melhorar desfechos que ainda são limitados. A fim de melhorar sintomas resistentes à ECP, como cognição e marcha, novos alvos cerebrais estão sendo explorados. Na presente revisão, discutimos o atual estado da arte relacionado à ECP, abordando seleção de pacientes, implante cirúrgico de eletrodos, e programação do dispositivo, além de explorarmos novos avanços em desenvolvimento.

## INTRODUCTION

Deep Brain Stimulation (DBS) is a valuable treatment option to improve quality of life in patients with Parkinson’s disease (PD)^1^. Several good quality studies have established the advantages of DBS over best medical therapy in carefully selected patients^1^ . However, there is still disinformation regarding DBS, both among health care providers, patients and caregivers, which contributes to the underuse of this therapy^2^. For example, a reasonable proportion of neurologists are not familiar with DBS indications, and often overestimate potential negative aspects of outcome, such as acute complications and side effects^2^, being a hurdle for potential surgical referral for many good DBS candidates. Beyond indication, the number of neurologists capable of adequately programming a DBS device is limited, leading to not infrequently finding patients inadequately programmed, exposed to stimulation side effects or suboptimal improvement of parkinsonian signs and symptoms. 

Although important advances were achieved in the DBS field throughout the last decades, new developments in patients’ selection, surgical approach, programming, target selection, and telemedicine are in the pipeline, with the capability to address current limitations.

In this review, we address the current state of the art of DBS in PD and discuss future perspectives to improve DBS therapy that are currently in development. 

## DBS IN PD: CURRENT STATE OF THE ART

### The “whos” and “whens” - indications, requirements, and timing of DBS surgery for PD patients

There are currently three indications for DBS in PD patients: refractory motor fluctuations/complications of chronic levodopa therapy (wearing off, dyskinesias, etc.), refractory tremor, and intolerance to dopaminergic agents^3^. In addition to indications, patients should also fulfill other basic requirements. The initial step is diagnosis of idiopathic PD, keeping in mind the risk of diagnosis error in PD, especially in the first years of symptoms^4^. As such, the second, almost intuitive requirement is the need for referral after more than four years since onset of motor symptoms^5^, limiting the risk of operating on patients with a different form of parkinsonism that recognizably does improve after DBS, such as atypical or secondary^6^ ^,^ ^7^. Another requirement is a minimum of 33% improvement in the Levodopa Challenge Test (LCT). In brief, this test measures the effect of a suprathreshold dose of levodopa by comparing Unified Parkinson’s Disease Rating Scale part III (UPDRS III) scores in two different conditions: “OFF-med” (12 hours withdrawal of dopaminergic medications) and “ON-med” (at the peak of a levodopa suprathreshold dose)^8^. The importance of this test relies on its ability to predict DBS motor response both qualitatively and quantitatively, probably reflecting connective integrity of structures outside the presynaptic nigrostriatal dopaminergic pathway^9^. Pragmatically, if the most bothersome symptoms are not responsive to levodopa, the patient might not be the ideal DBS candidate, except for refractory tremor. Although the arbitrary cut-off of 33% is per se an indicative of acceptable motor response, improvements beyond this benchmark indicate a proportionally better response after DBS^10^. The fourth requirement is absence of significant cognitive deficits or uncontrolled neuropsychiatric diseases, evaluated with neuropsychological and neuropsychiatric batteries^11^. It is important to highlight that mild cognitive impairment warrants the need to tread lightly but does not necessarily exclude the possibility of DBS surgery. Finally, patients must be able to attend frequent medical appointments after surgery, to accurately take medications, to follow a rehabilitation plan, and deal with potential, although infrequent, surgical complications such as intracranial hemorrhage and infection; in other words, patients need a minimum of psychosocial independence and / or support. 

## OUTCOMES AND SIDE EFFECTS

When referring PD patients to DBS surgery, physicians, patients and caregivers must be aware of the expected motor and non-motor outcomes. Regarding motor symptoms, based on current predictions of outcome, it is expected that PD patients improve a mean of 50% in UPDRS III scores at six months to two years after surgery^10^. More specifically, one year after DBS, tremor improves by a mean of 74%, rigidity improves by 57%, and bradykinesia by 49%^1^. Dyskinesias are expected to improve a mean of 80% in duration, and 94% in disability one year after DBS^12^. Axial symptoms, despite reaching a mean improvement of up to 57% during the first year after surgery, are expected to be 6% worse after eight years compared to preoperative evaluation, which can be explained by the ongoing progression of the underlying degenerative process^1^. 

Selected non-motor symptoms, although not typically improved by treatment in PD patients as a rule, can improve after DBS. Pain, sleep, and behavioral non-motor fluctuations can be improved^13^, while antiparkinsonian medication regimen can be reduced and made more flexible after DBS, especially when the subthalamic nucleus (STN) is the target of choice^1^.

Beyond motor and non-motor outcomes, it is paramount that both patients and physicians realistically recognize possible complications of DBS surgery. In terms of immediate complications, intracranial hemorrhage (symptomatic or not) is reported in 4,4% of cases^2^. These chances are lower (down to 0,7%) when surgery is performed in more experienced medical centers^2^. The risk of symptomatic bleeding leading to permanent deficits is in the range of 1.6%^10^. Infection, the most common surgery-related complication, can occur in 5.1% of cases^10^. During the period following the procedure, weight gain is the most common adverse event after DBS, reported in 36%, probably due to a combination of factors, including dyskinesia improvement, behavioral changes, etc.^1^. Another important side effect is change in speech intelligibility with a combination of dysarthria and hypophonia, which can happen in about 20% of patients. It is important to highlight that these speech changes after DBS might be secondary to the surgery itself (adverse lesional effect), to disease progression (unrelated to DBS), or to electrical current spread to speech related tracts. The latter is potentially addressed in part by different techniques of DBS programming. Currently, DBS surgery is considered cognitively safe when a thorough preoperative assessment protocol deems patients eligible^14^. 

## BRAIN TARGET SELECTION

For decades, there has been heated debate regarding the best brain target for DBS in PD. This discussion concerns the STN and globus pallidus internus (GPi) for the most part, as the ventral intermediate nucleus of the thalamus can improve PD tremor, but does not improve other parkinsonian symptoms, such as bradykinesia, rigidity, and dyskinesias^13^. Overall, STN-DBS has been the preferred target by many centers as the one with the largest body of evidence for a superior outcome in broad terms^10^. On the other hand, comparative studies between STN and GPi DBS often fail to provide undisputable and sizable superiority of either target on motor and non-motor outcomes^10^. In most instances, STN-DBS allows for medication reduction, with a mean decrease of 50% in antiparkinsonian medications one year after surgery^1^ ^,^ ^10^. The mean reduction of dyskinesias is similar among the targets: after STN-DBS ranges between 20 and 83%, while after GPi-DBS between 40 and 87%^10^ ^,^ ^15^. GPi-DBS appears to be safer for older, more frail, mild cognitively impaired and brittle dyskinetic patients^16^. However, some authors argue that the ability to reduce antiparkinsonian medications with STN-DBS, particularly dopamine agonists, could also play a positive role for patients with certain profiles of cognitive and behavioral issues^17^. The choice of brain target can be complex, and should fall under the umbrella of precision medicine, being discussed on an individual basis by all parts involved in these challenging cases of care. 

## HOW TO PREDICT CLINICAL RESPONSE AFTER DBS?

Bearing in mind that DBS surgery is costly, and not without risks, many studies have investigated preoperative clues that could refine criteria for eligibility and predict good or bad response^18^. To date, one of the most important predictive factors for good response to DBS surgery is the LCT^8^. Numerous STN-DBS randomized controlled trials report a linear correlation between improvement in UPDRS III after surgery, and improvement in the LCT^10^. It is important to highlight that this correlation is also qualitative; in other words, symptom specific: for example, in a patient with gait problems, if gait improves on the LCT, it should likely also improve after DBS. Another important predictive factor is age. Patients with younger onset PD, and patients younger at time of DBS surgery tend to have longer-lasting and more robust motor outcomes^19^. Cognitive status also typically reflects greater functional improvement after DBS^19^, probably reflecting the fact that cognitively preserved patients are more capable of appreciating and taking advantage of the positive effects of surgery. Recent studies similarly correlated quality of life with DBS outcomes. The worse the preoperative quality of life, the larger its improvement 24 months after DBS, corroborating the notion that patients need to be significantly impacted by the disease before being considered DBS candidates^20^. Since dysarthria is one of the most common side effects after DBS, it is important to have in mind that poor preoperative speech intelligibility and longer disease duration are predictors of deterioration of speech after STN-DBS^21^. 

## HARDWARE EVOLUTION: THE NEW KIDS ON THE BLOCK

During the past decade, the DBS field evolved considerably not only from a technological standpoint. Today, the design of implanted pulse generators (IPG), which are usually implanted in subcutaneous thoracic wall region and provide energy for DBS functioning, evolved compared to older models, with rounded edges and considerably smaller sizes, which are advantageous as bulky and square-edged IPG are prone to skin rupture, infections and discomfort, especially in patients with lower body mass indexes. Additionally, patients can choose between a rechargeable (smaller, recharged at home, around once weekly) and non-rechargeable IPG (slightly larger, no need to recharge, replaced approximately five-yearly). Finally, almost all currently available IPG are compatible with magnetic resonance imaging (MRI), therefore, the anticipation of future MRI need is no longer a variable to be weighted in when referring patients for DBS surgery. 

The success of DBS relies not only on appropriate candidate selection, but also on strict and precise surgical technique^22^. In this regard, an important new feature, directional electrodes, allows for a more precise delivery of the electric field when electrodes are subtly misplaced. In these devices, two out of four contacts are divided into three radial subdivisions allowing for current steering directed to the intended target structures, avoiding, at the same time, stimulation of adjacent structures that can provoke side effects. In other words, this innovation broadens therapeutic windows^23^. 

Multiple independent current control (MICC), a new technology that allows separate current controls for each electrode contact, opens the possibility of exploring independent current variables in different contacts within the same electrode. Also, physicians can distribute current strength differently throughout contacts. The combination of MICC with directional leads allows for better current malleability and facilitates reaching targeted structures more efficiently^24^.

Beyond its clinical use, hardware advances in DBS have also helped for a better understanding PD pathophysiology. New brain-sense devices (Percept PC^TM^, Medtronic, USA) can continuously record local field potentials (LFP). LFP represent the “electrical signature” of the neuron population around a given contact. It is known that LFP bursts of activity in the beta band (13 - 30Hz) correlate with PD bradykinesia and rigidity^25^. Both levodopa therapy and DBS can effectively reduce beta bursts while improving parkinsonian symptoms. Therefore, beta bursts can be used as biomarkers of akinetic-rigid parkinsonian signs^26^. Likewise, bursts of gamma activity (30 - 200Hz) are linked to dyskinesias^27^. When adding LFP recording to DBS programming, physicians have access to yet another parameter that can aid in patient monitoring outside the clinic and finding the best electrode configuration. Even further, optimization of brain sense technology can be the first step for closed-loop DBS, in which stimulation can be tailored according to the changing needs of patients throughout the day^28^. 

## CURRENT PROGRAMMING STRATEGIES

Even when a PD patient is correctly selected for DBS and the electrode is well-placed, suboptimal programming can limit effectiveness and expose patients to stimulation induced adverse effects that could otherwise be readily addressed. Therefore, it is paramount that clinicians involved in DBS therapy are qualified to proper and personally program these devices and explore their innovative features. 

Most DBS patients will sufficiently improve with monopolar stimulation, in which one contact is used as cathode while the anode is the IPG. The best contact (cathode) must be identified for each hemisphere after a thorough monopolar review^29^. This configuration should always be tried first as it is more energy efficient and able to explore the target in its entirety. 

However, not infrequently, different configurations need to be explored. In bipolar stimulation, when one contact is the cathode and another is the anode, there is less current spreading to adjacent areas. As such, this type of stimulation is useful when the intended target is a small area around the electrode or when monopolar stimulation has a tight therapeutic window (high threshold for therapeutic effect and/or low threshold for adverse effects)^29^. On the other hand, when the intention is to reach larger areas (i.e., GPi), double monopolar configuration, in which two contacts are used as cathodes while the IPG remains as anode, can be used. This configuration, however, is rarely used in small targets such as the STN due to the risk of current spreading to unwanted structures^29^. Another form of stimulation is interleaving, or multistim, in which two programs quickly alternate^30^. This should be attempted 1) when two contacts are used to improve two distinctive clinical features with different therapeutic windows and electrical variables (except for frequency), 2) to avoid current-spreading side-effects, 3) to increase stimulation frequency in a small overlap area while keeping lower frequencies at the core of the individual electric fields, and 4) to reach a larger target area without the risk of adverse effects seen with double monopolar stimulation^30^. It is important to remember that all these types of alternative programming demand more energy consumption, shortening battery life compared with monopolar stimulation.

Compared to appendicular symptoms, axial symptoms such as speech, gait, and balance are less responsive to DBS. Several studies have examined the effects of low frequency stimulation (LFS, frequencies below 100Hz) in axial parkinsonian symptoms. LFS can improve speech, gait, and balance in some patients and this improvement can be enduring^31^. However, LFS seems to better improve axial symptoms when these symptoms are induced or worsened by standard high frequency stimulation (HFS). Furthermore, even when the improvement in axial symptoms is observed, some patients cannot withstand LFS due to worsening of appendicular symptoms, particularly tremor. In these cases, a strategy using interleaving stimulation with two LFS programs in adjacent contacts can be tried as it induces an overlap of the electric fields, generating an area of HFS (namely, double the individual frequency of the interleaved programs)^32^. Short pulse width (pulse widths below 60µs) is non-inferior to longer pulse width stimulation in lowering UPDRS III score, is able to increase the therapeutic window, and reduce battery usage, but may fail to improve speech problems in PD patients^33^. Other programming strategies to improve gait in PD can be tried, particularly when the gait parameters are asymmetric or when freezing of gait (FOG) is clearly driven by one body side. In these conditions, improvements can be obtained when current is reduced contralateral to the side of larger step length^34^, or when LFS is used contralateral to the side in which FOG most usually occur^34^. The combined stimulation of STN and substantia nigra (SN) can also be tried to improve resistant axial motor impairment in PD patients^34^. 

## CURRENT LIMITATIONS OF DBS IN PD

Despite advances in technology, several symptoms are not responsive or are only transiently responsive to conventional DBS, and there is a critical need for improvements in the current DBS model. Although the LCT is the most used predictive factor for DBS in PD, it cannot properly assess tremor response after surgery and does not take into consideration medication-induced dyskinesias^10^. It can also be challenging to perform this test in patients with severe levodopa intolerance. Axial symptoms are often resistant to DBS, despite the use of the techniques already described in this review. Such problems, even when paired with good control of appendicular symptoms, critically impact quality of life and survival of PD patients^1^. New targets are still being investigated to address these issues, but so far, no concrete results were able to change our clinical practice. While BrainSense technology seems the first step to a closed-loop stimulation, in which DBS energy would be tailored to individual needs while avoiding over-stimulation, there are still many obstacles to overcome: 1) beta-band activity, although a biomarker for rigidity and bradykinesia, does not correlate so well with tremor and seems to naturally decrease with chronic DBS stimulation, 2) in many PD patients, there is no clear beta peak that can be tracked, 3) while current devices are able to track beta activity, there is still much to accomplish regarding signal processing to exclude signal artifacts, and 4) we are still unable to dynamically adapt energy according to patient’s needs. Surgical planning is another important limitation, since it cannot assure proper electrode placement in many cases, leading to suboptimal improvement after surgery. Finally, remote DBS programming is not yet widely available, which can prevent patients living far from DBS centers from receiving this powerful therapy. 

## ADVANCES IN DBS TECHNOLOGY

### How can brain image and genetics help in a patient’s selection?

The largest study to date about long term predictive factors for DBS correlated brain MRI vascular changes and motor improvement 1 and 10 years after surgery^18^. Thicker frontal cortical thickness also predicts better motor outcomes after STN DBS, with lower amplitude requirements for similar motor performance^35^. Preoperatory higher parieto-occipital glycolytic uptake as well as lower primary motor cortex glycolytic uptake also correlated with better motor improvement^36^. 

Regarding genetic forms of PD, important differences were observed according to the mutation type. Patients carrying the G2019S variation in leucine-rich repeat kinase-2 (LRRK-2) gene have substantial daily living activities improvement, but results are poorer with the rarer R793M and R1441G variant of this same gene, while homozygous and heterozygous PRKN mutation carriers have good outcomes and minimal cognitive decline up to five years after STN-DBS surgery. Glucocerebrosidase (GBA) gene mutation carriers, however, have lower mean medication dose reduction, and significantly worse cognitive and neuropsychiatric outcomes after DBS^37^. Longer follow up studies found ten times more severe cognitive impairment in GBA after surgery^37^. Data is scarce or absent for drawing substantial conclusions in other genotypes.

The evolution of genome wide association studies allowed for the exploration of clinically less significant single nucleotide polymorphisms in large groups with promising findings^37^, and soon genetic exploration might become a valuable tool for patient, target, and therapy selection. 

## TARGET VISUALIZATION AND PROGRAMMING ACCURACY (IMAGE-BASED)

In DBS surgery, millimetric accuracy is crucial to minimize error in lead placement (38). Several factors contribute to inaccuracies of stereotactic procedure, including quality of pre-operative and intraoperative imaging^38^ ^,^ ^39^. 

Preoperative patient-specific MRI (direct targeting), standardized atlases (indirect targeting) or hybrid targeting aid in optimal target location^40^. The feasibility and accuracy of direct targeting is mainly dependent on the quality of the MRI^38^ ^,^ ^41^ ^,^ ^42^. Most targets used in functional neurosurgery are suboptimally visualized on routine low field 1.5 Tesla or 3 Tesla MRI^43^ ^,^ ^44^. Advances in neuroimaging technology over the past decades may overcome this limitation with the availability of ultra-high-field (UHF)-MRI and the use of new sequences resulting from changes in MRI acquisition parameters^38^ ^,^ ^43^ ^,^ ^44^. Low field MRI is associated with limited contrast and signal to noise ratios and generates images that lack sharp and clear borders for small deep brain structures^42^ ^,^ ^45^. UHF MRI systems (7T and above) can obtain submillimeter anatomical information^46^. The main benefit of UHF-MRI is the increase in signal-to-noise ratio (SNR), which allows increased spatial resolution, facilitating visualization and delineation of smaller neuroanatomical structures, reducing the gap between MRI and histology^44^ ^,^ ^46^. This is particularly relevant in DBS planning, since SNR scales inversely with distance from the cortex^44^. UHF MRI is also associated with a better contrast-to-noise ratio (CNR) providing better differentiation between small abutting structures^46^. Increased susceptibility artifacts related to the UHF may be an advantage in some cases. It improves visualization of iron-rich structures like the STN. Moreover, it would allow direct identification of nuclei not visible in lower field MRI. Kanowski et al.^47^ reported that thalamic subfields were successfully delineated in the dorsal aspect of the lateral thalamus with the use of 7T MRI. However, UHF MRI has disadvantages, including higher susceptibility to distortions, safety concerns related to metallic implants and reduced availability. 

Besides increases in magnetic field strength, alternative MRI sequences have also improved image quality, allowing direct targeting^38^ ^,^ ^43^ ^,^ ^44^ ^,^ ^48^. Usually, MRI sequences can be separated into spin echo (SE) methods and susceptibility-based sequences. The first group includes T2 weighted imaging and inversion recovery, sequences often poor in precise targeting. The second group includes SWI, T2*WI, and considers differences in brain tissue composition. Limitations of this group are signal loss, distortion and local field heterogeneity which can blur the edges of the target^48^ ^,^ ^49^. 

O’Gormann et al. analyzed the optimal MRI methods for direct stereotactic targeting of STN and GPi, and observed that SWI offers the highest CNR for the STN, but standard proton density weighted (PD-W) images provide the best CNR for the pallidum^49^. Sudhyadhom et al., described the Fast Gray Matter Acquisition T1 Inversion Recovery (FGATIR) 3T MRI and pointed that this technique allows thalamus, striatum, GPi, red nucleus, and substantia nigra localization, and displays sharper structure delineation. Proton density is a modern sequence that reflects density of protons in tissues and provides excellent contrast between white and gray matter structures making it useful to target GPi and pedunculopontine nucleus^44^. 

Quantitative Susceptibility Mapping (QSM) is a novel image processing technique that can be applied to multi-echo GRE acquisitions^48^. It quantifies the susceptibility in each structure and represents them on a scale that enhances the contrast between neighboring structures. QSM goals include reducing orientation dependency of the targeted brain tissue, thus diminishing blooming artifacts, and providing a more direct measurement of intrinsic tissue magnetic properties^44^. Rasouli et al. reported that targeting STN using QSM can be safely used for DBS lead placement with satisfactory clinical response^50^.

**Figure 1.**
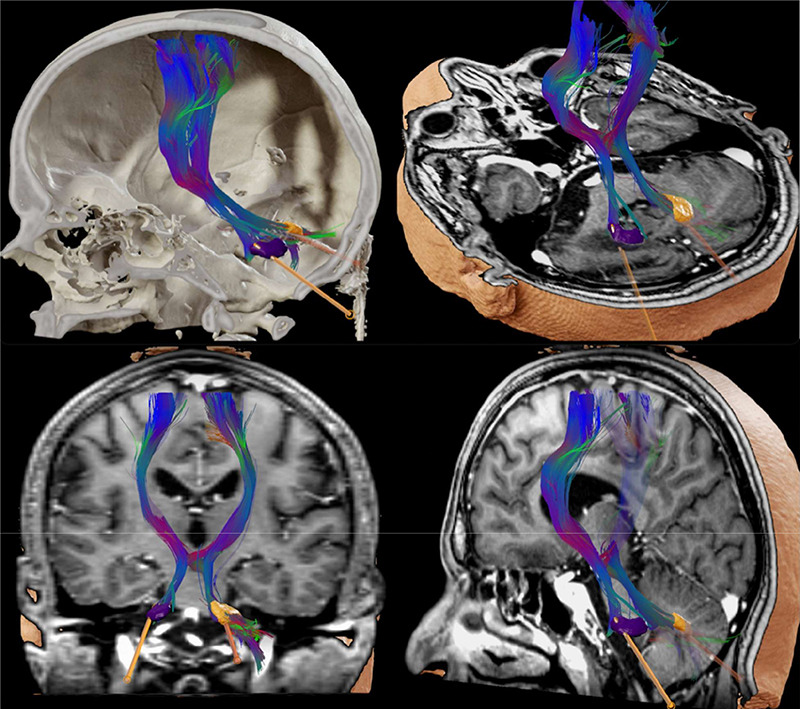
Three-dimensional reconstruction of DBS electrodes in bilateral dentate nucleus - Dentatorubrothalamic Tract was reconstructed and used to refine lead position. The 3D reconstructions and tractographies were performed with Elements software (Brainlab AG, Germany)

Diffusion weighted imaging and tractography are also gathering interest as a targeting tool focusing on white matter tracts. It is accepted that DBS can have influence over widespread areas of the brain, which have implications beyond the inhibition of a local gray matter structure^51^. One of DBS mechanisms is modulation of circuit activity and fiber pathways in the vicinity of the electrodes. These image techniques offer unprecedented visualization of brain connections relevant to DBS safety and efficacy^38^. King et al. reviewed studies analyzing the use of DTI for DBS surgical planning, and concluded that it provides additional information over conventional targeting methods, and can improve outcomes^51^. This technique not only identifies fibers relevant to DBS targeting, but also delineates a more conventional target, suggests how modulation of these pathways lead to improved outcomes, allows differentiation of targeted fibers from those associated with side effects, and supports a more individualized stimulation^44^ (Figure 1).

**Figure 2.**
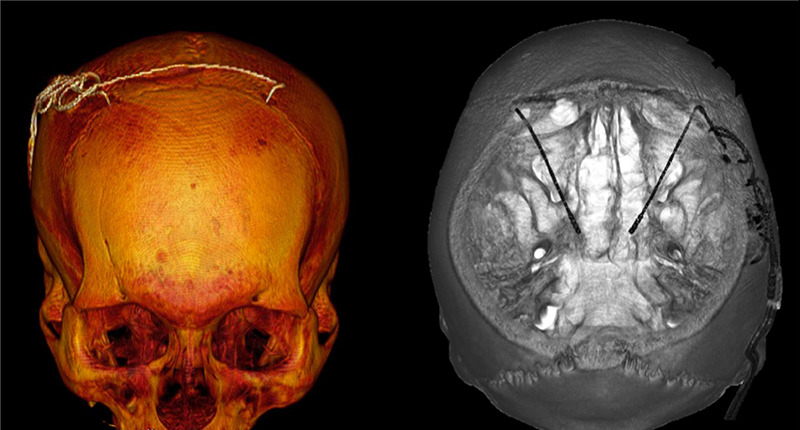
Three-dimensional reconstruction of DBS electrodes using an open-source software.

**Figure 3.**
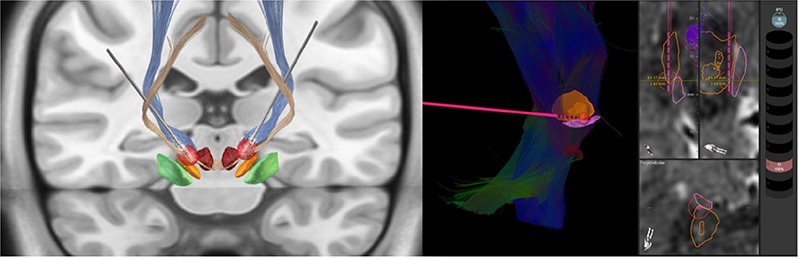
A. Three-dimensional reconstruction of DBS leads exposing bilateral Volume of Tissue Activated in red. Reconstruction performed with Lead DBS software. B. Three-dimensional reconstruction of DBS lead evidencing Volume of Tissue Activated in red. Reconstruction performed with Elements software-Guide XT.

Several software tools have been introduced to improve lead position visualization (Figure 2). Lead-DBS provides DBS electrode placement based on pre-and postoperative MRI or computed tomography, and electrodes’ relationship to surrounding brain areas. It also provides Volume of Tissue Activated (VTA) visualization, estimating the region activated by electrical stimulation based on a patient’s stimulation parameters^43^ (Figure 3). Reconstruction of precise electrode placements relative to surrounding anatomical structures is particularly helpful during programming, especially with directional leads. Until recently, DBS programming was mainly based on clinical response testing, a time-consuming task. Therefore, placement reconstruction can decrease programming time, and allows a more standardized approach that could potentially reduce inter-rater variability^2^ ^,^ ^43^. 

Unlike diagnostic radiology, imaging for stereotactic surgery requires more than just visualization of structures. Together these neuroimaging advances aim to improve stereotactic targeting for awake and asleep DBS, reduce operation time, optimize DBS programming, and address the question of a more personalized stimulation.

## NOVEL STIMULATION PATTERNS

**Figure 4.**
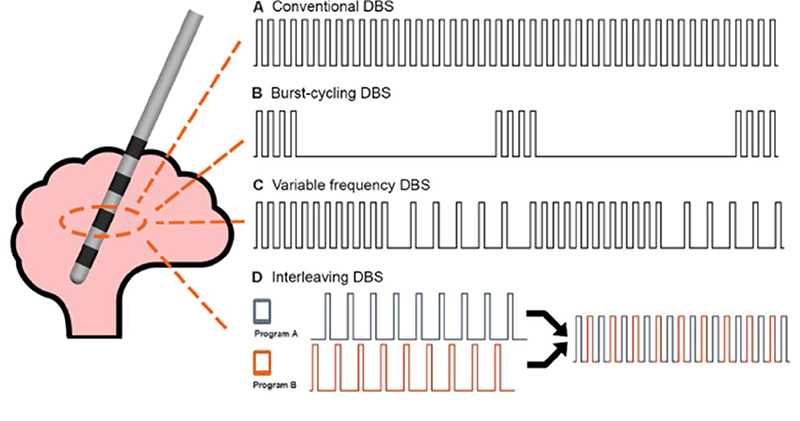
Exploratory stimulation patterns A. conventional DBS, B. Burst-Cycling DBS, C. Variable frequency DBS, and D. Interleaving DBS. Adapted from Wong JK, Hu W, Barmore R, Lopes J, Moore K, Legacy J, et al. Safety and Tolerability of Burst-Cycling Deep Brain Stimulation for Freezing of Gait in Parkinson’s Disease. Front Hum Neurosci. 2021 Apr 26;15:651168^52^.

The stimulation waveform and patterns themselves have been subjected to scrutiny for optimization (Figure 4). Anodic stimulation appears to produce a larger therapeutic window but with disproportionally higher energy usage^53^. In variable frequency stimulation, there are cycles of at least ten seconds of high (>130Hz) and low (60-80hz) frequencies on a single contact. Initial studies yielded positive results with better motor outcomes and significant improvement of FOG, but larger trials are lacking^54^. Burst-cycling stimulation, in which regular frequency stimulation is administered in intermittent bursts, appears to be more energy efficient with similar clinical outcomes^55^. Symmetric biphasic pulses can apparently lead to better motor improvement, but with greater battery drainage^56^. 

## INTELLIGENT DBS

The current approach for DBS programming is an “open loop” model, with continuous stimulation parameters left unchanged between periodic adjustments. However, patients’ needs are dynamic. In closed loop stimulation, or adaptive DBS (aDBS), programming is constantly adjusted according to biomarkers correlating to patients’ symptoms^57^.

Biomarkers can be external, such as wearable devices (finger motion sensors, wristwatches with accelerometers), or internal (surface electromyography, cortical sensing, or basal ganglia recording of LFP)^58^ ^-^ ^62^. 

As mentioned above, beta band oscillations (11-35Hz) in basal ganglia LFP are well correlated with off symptom intensity^25^ and can be suppressed by medication and DBS^58^. aDBS trials using beta band as a biomarker demonstrated reduction by half on stimulation time with better motor outcomes than conventional stimulation^59^, including less dysarthria^60^ and less severe dyskinesias^61^.

Another viable biomarker for aDBS can be obtained from cortical recordings. Gamma band activity (60-90Hz) has little interference from movement and is a good marker of dyskinesia, explored successfully with sustained clinical benefit in two patients^62^.

Current aDBS approach still requires most of the programming to be prepared by the clinician, and not all symptoms correlate well with each specific biomarker. The strategy will likely have to shift to a multiple input paradigm suitable for broad use among most patients.

## NEW TARGETS

Although dopaminergic medications and DBS of the conventional targets (STN and GPi) significantly improve cardinal symptoms in PD, effects on gait and balance are less consistent and not well sustained in the long-term. Additionally, cognitive functions are usually not affected by or can even worsen after stimulation. In this context, recent trials have attempted to modulate alternative regions to tackle these axial and cognitive symptoms^63^.

### Substantia nigra pars reticulata (SNr)

SNr is a primary output nucleus of the basal ganglia that sends GABAergic projections to the pedunculopontine nucleus. In PD, the SNr is abnormally overactivated, which inhibits the locomotor region and contributes to gait problems observed with disease progression^63^. Double stimulation of the SNr and the STN was superior in controlling FOG compared to STN stimulation alone^34^. Another recent study showed that high-frequency stimulation of the SNr but not of the STN improved the anticipatory postural adjustments in PD, confirmed by two other trials^63^. 

Although promising, few patients have been included, and there are still uncertainties regarding the best stimulation parameters and the hot spot of stimulation inside the SNr to improve locomotion.

### Spinal cord

In the last years, spinal cord stimulation (SCS) has been suggested to improve axial symptoms in PD patients, especially gait and posture abnormalities^64^. An open-label study including 15 PD patients reported improvement in postural instability and gait speed over 12 months of follow-up^66^. Another open-label study demonstrated improvements in several gait parameters after thoracic SCS in five PD patients during 6 months of follow-up^67^. More recently, an open-label study with 6 PD patients failed to show any benefit 12 months after thoracic SCS^68^. 

Despite good results of SCS in treating gait problems, only a small number of PD patients were evaluated so far, with variable study populations. Furthermore, the stimulation produces tangible sensations which might be responsible for a placebo effect, which should be addressed in newer trials^69^. Double-blind approaches designed with an amplitude subthreshold for paresthesia, very high frequencies (below the sensory threshold), or new paradigms such as burst stimulation, will hopefully guide these future trials to avoid placebo effects^70^. 

### The nucleus basalis of Meynert (NBM)

The nucleus basalis of Meynert (NBM) is largely involved in many cognitive functions, including arousal, attention, perception, and memory and its stimulation has recently emerged as a potential new therapeutic option in PD patients with mild cognitive impairment or dementia^71^.

To date, there are five case reports and four randomized crossover studies involving patients with PD with dementia and Lew body dementia (LBD)^72^. Although DBS seems to be safe and well tolerated, no significant improvement of cognitive scores between sham vs. active NBM DBS has been detected.

Although the primary outcomes were not achieved on these trials, decreased neuropsychiatric scores, which was primarily driven by a reduction of visual hallucination and apathy, were noticed in some patients. Moreover, improvement of functional connectivity in LBD subjects was also observed^73^. More preclinical evidence is needed to optimize NBM DBS, such as patient selection and DBS parameters. The addition of in vivo cholinergic imaging might contribute to understanding the mechanism of NBM modulation and its influence on brain connectivity.

## DBS AND TELEMEDICINE

The use of telemedicine, although not new, has greatly expanded since the COVID-19 pandemic. In some cases, basic technological barriers can prevent patients from being properly assessed with this tool. However, if patients have stable internet access, are comfortable with technology, have caregiver support, and have a compatible device, telemedicine can be an option to reduce traveling time, especially in large countries^74^. In PD, it is especially relevant, since tremor, bradykinesia, gait, and nonmotor symptoms can be assessed through videoconference, enabling access to specialized care for patients living in distant areas. This is particularly important for advanced PD therapies, since DBS indication, implantation, and programming should ideally be done in referral centers. Although the technology for remote programming of cardiac pacemakers is widely available, DBS remote programming is still under development. To date, all DBS manufacturers allow for group programming, in which different DBS configurations can be set as groups, and patients can change groups in their homes using patient’s controllers. Also, if previously set, patients can make small changes in the stimulation amplitude. However, this type of remote programming available today is very limited. In March 2021, the U. S. Food and Drug Administration approved Abbot Labs (Plano, TX, USA) Neurosphere^TM^ Virtual Clinic, a new functionality of the Infinity^TM^ DBS systems that allows remote DBS programming^74^. There are also two Chinese DBS manufacturers testing remote programming^74^. However, this technology is not yet worldwide available. 

In conclusion, to benefit more PD patients, realistic information about DBS outcomes, patient selection, and adequate programming need to be spread to general neurologists that are still unfamiliar with this treatment. Reluctance in surgical referral, inadequate indications, suboptimal lead placement, and poor programming skills prevent patients from achieving the best possible outcomes after surgery. Moreover, there are still many other limitations that need to be addressed regarding DBS therapy, and many important questions remain unanswered. Continuous innovation and new studies on unexplored facets of this ever-growing field are currently expanding the frontiers and potential achievements of this powerful therapy.
